# Surgical repair of large adult Bochdalek hernia: case series and literature review

**DOI:** 10.3389/fsurg.2025.1713049

**Published:** 2026-01-08

**Authors:** Shahd Abbastanira, Omar Almarzouqi, Sura Al rawi, Samah Kannas, Sara Bashier, Hala Mrayyan, Labib Al Ozaibi

**Affiliations:** 1 Graduate Medical Education, Mohammed Bin Rashid University of Medicine and Health Sciences (MBRU), Dubai Health, Dubai, United Arab Emirates; 2 General Surgery, Consultant Surgeon, College of Medicine (CoM), Mohammed Bin Rashid University of Medicine and Health Sciences (MBRU), Dubai, United Arab Emirates

**Keywords:** bochdalek hernia, diaphragmatic hernia, surgical repair, adult, case report

## Abstract

**Introduction:**

Bochdalek hernia is a rare congenital diaphragmatic defect, typically diagnosed in infancy. Adult presentation is uncommon, and management remains debated, particularly in asymptomatic cases. This report presents two adult cases successfully treated with surgical repair, contributing to the limited literature on adult Bochdalek hernias.

**Presentation of case:**

A 20-year-old male, with no prior medical history, was incidentally found to have a left posterolateral diaphragmatic defect (5 × 7 cm) containing small bowel, splenic flexure of the colon, and the left kidney. Laparoscopic repair was attempted but converted to open surgery due to limited visualization and tension. Postoperatively, he developed a left pneumothorax and pneumoperitoneum requiring chest tube placement and was discharged on day six. A 43-year-old female with previous gastric banding presented with recurrent nausea, vomiting, and abdominal pain. Imaging revealed a large left diaphragmatic hernia (12 × 6 cm) containing stomach and bowel segments. She underwent emergency open repair. Postoperative recovery was complicated by a minimal left pneumothorax, which resolved, and she was discharged on day nine.

**Discussion:**

Adult Bochdalek hernias are rare and may be discovered incidentally or present with acute symptoms. Surgical repair is recommended to prevent complications, and laparoscopic approaches may require conversion to open repair in cases of large defects or high tension.

**Conclusion:**

Early recognition and timely surgical intervention are crucial for adult Bochdalek hernia, even in asymptomatic patients. These cases highlight effective management strategies and enhance understanding of this rare adult presentation.

## Introduction

Congenital diaphragmatic hernia (CDH) is a rare birth defect, occurring in approximately 1 to 5 per 10,000 live births. It results from incomplete formation of the diaphragm, allowing abdominal viscera to herniate into the thorax, potentially causing respiratory insufficiency and persistent pulmonary hypertension (1). CDH has multiple variants, with Bochdalek hernia being the most common, accounting for 80%–90% of cases (2). Although diaphragmatic hernias have been documented since 1,690, Bochdalek first described the posterolateral developmental defect in 1,848 (3). Less common variants include Morgagni hernia (anterior diaphragm), central diaphragmatic hernia, or complete absence of the diaphragm replaced by a thin membrane (2).

CDH is typically diagnosed in neonates and young children; with advances in prenatal imaging, most cases are now identified during the second trimester (18–24 weeks) using ultrasound (4). Adult presentation is extremely rare, with incidental Bochdalek hernia reported in only 0.17% of cases (3). Fewer than 200 cases of occult Bochdalek hernias in asymptomatic adults have been documented in the literature (5).

This case series presents two distinctive adult cases of Bochdalek hernia: one entirely asymptomatic and the other presenting with obstructive symptoms.

## Case presentation

### Case 1

A 20-year-old male with no known medical, surgical, or family history presented to the outpatient general surgery clinic following the incidental detection of a diaphragmatic hernia. The hernia was initially identified on a chest x-ray performed at a private facility as part of a mandatory medical check-up for his employment in the armed forces. He denied any symptoms, including abdominal pain, dyspnea, gastrointestinal disturbances, or history of trauma.

**Figure 1:**
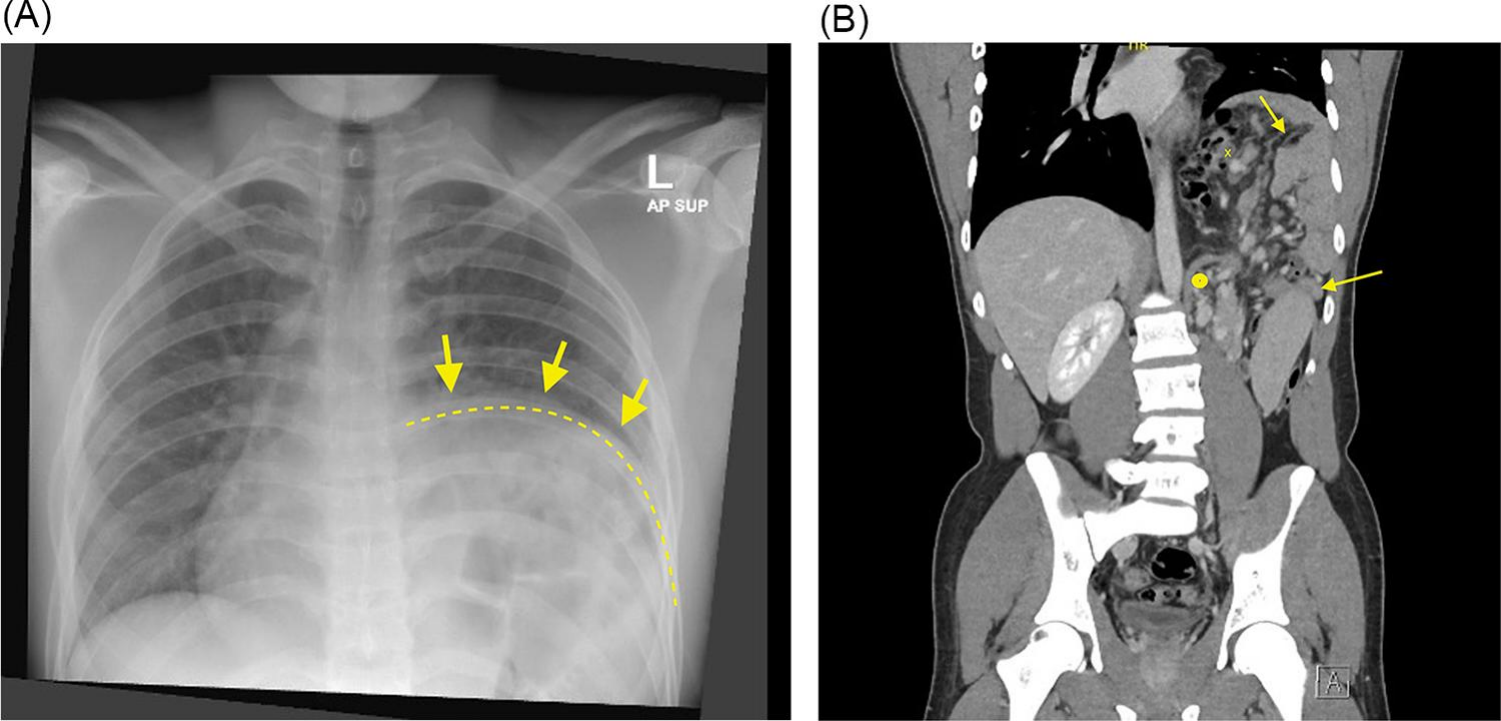
**(A)** Chest x-ray, AP view. Showing a large left diaphragmatic hernia with abdominal contents in the left hemothorax (arrows), and a mediastinal shift towards the right side. **(B)** Preoperative CT scan of the chest, abdomen, and pelvis with contrast, coronal view. Showing a left posterior defect on the left hemidiaphragm with herniation of small bowel loops, splenic flexure of the colon and left kidney with a hernia defect measuring about 5 × 7 centimeter (**Arrow**; splenic flexure of the colon. **X**; small bowels, *; left kidney).

On physical examination, he was hemodynamically stable. His abdomen was soft and non-tender, with no palpable masses. Respiratory examination revealed decreased breath sounds at the left lower lung field. Chest x-ray revealed a large left diaphragmatic hernia with abdominal contents herniating into the left hemithorax, causing mediastinal shift to the right (Figure 1A). Contrast-enhanced CT of the chest, abdomen, and pelvis demonstrated a left posterior diaphragmatic defect measuring approximately 5 × 7 cm, with herniation of small bowel loops, the splenic flexure of the colon, and the left kidney. The left lung volume was reduced with mild ground-glass opacities in the left lower lobe. No evidence of bowel obstruction or ischemia was noted (Figure 1B).

The diagnosis of a large left diaphragmatic hernia was established through imaging. The incidental and asymptomatic nature of the hernia posed a challenge regarding the timing and urgency of surgical intervention. A multidisciplinary surgical team planned a laparoscopic diaphragmatic hernia repair; however, intraoperatively, limited visualization and tension led to conversion to open surgery. The herniated contents, including bowel and the left kidney, were successfully reduced, and the diaphragmatic defect was repaired primarily. A left subcostal incision was made, and dissection was continued until the remaining lateral diaphragmatic defect was identified. The defect was closed by approximating the edges to the adjacent ribs to ensure a stable repair. A 12 cm Symbotex® composite mesh was then applied and secured with non-absorbable sutures. A thorough small bowel run confirmed viable loops with no evidence of ischemia. The procedure was performed under general anesthesia, with a total operative time of approximately seven hours, and estimated blood loss was 150 mL.

**Figure 2:**
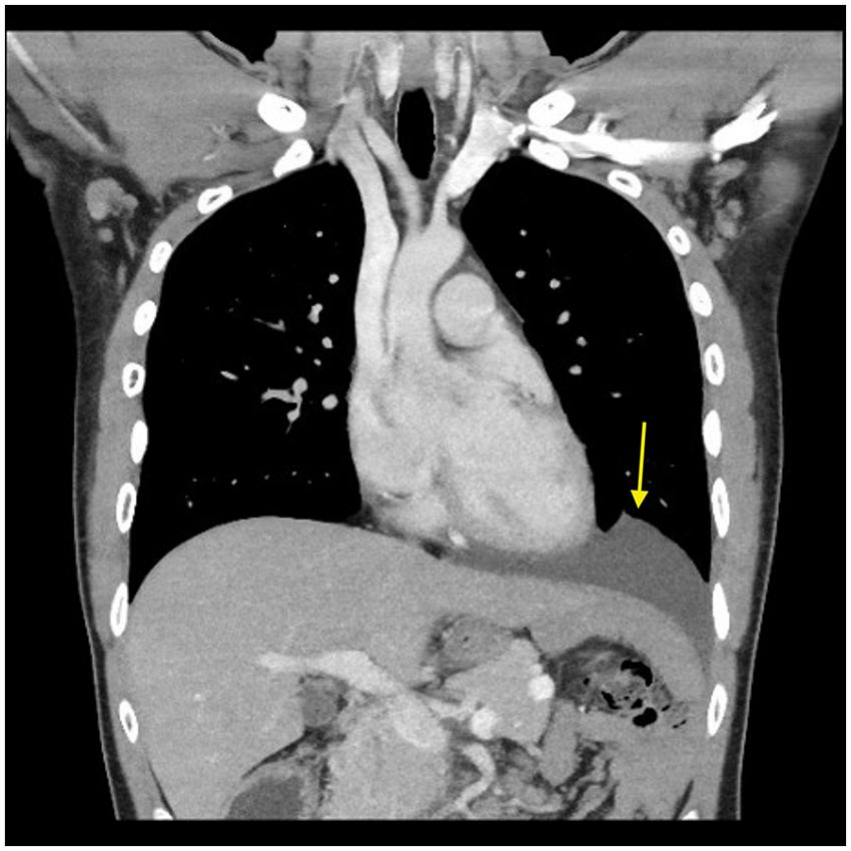
Postoperative CT scan of the chest, abdomen, and pelvis with contrast, coronal view. Showing reduced previously herniated abdominal organ into the left hemidiaphragm (arrow).

Postoperatively, the patient developed a left pneumothorax and pneumoperitoneum requiring chest tube insertion. He recovered well, resumed normal function, and was discharged in stable condition on postoperative day six. Follow-up CT demonstrated complete reduction and resolution of herniation (Figure 2). At one-month follow-up, he reported no symptoms and had returned to normal daily activities.

### Case 2

A 43-year-old female with past surgical history of gastric banding in 2007 for obesity management presented to the Emergency Department with recurrent upper abdominal pain, nausea, vomiting, and a burning sensation worsening over the preceding twenty-four hours. Two weeks prior, she had noted intermittent upper abdominal discomfort associated with postprandial bloating and nausea, and occasional blood in the stool. She denied other gastrointestinal symptoms, chest pain, or trauma. Her past surgical history was significant for laparoscopic gastric banding in 2007, which was initially uneventful but later complicated by band erosion and a localized foreign body reaction requiring corrective laparotomy in 2007, during which the band was removed and the gastric wall repaired. She had a midline laparotomy scar. Family history was non-contributory.

**Figure 3:**
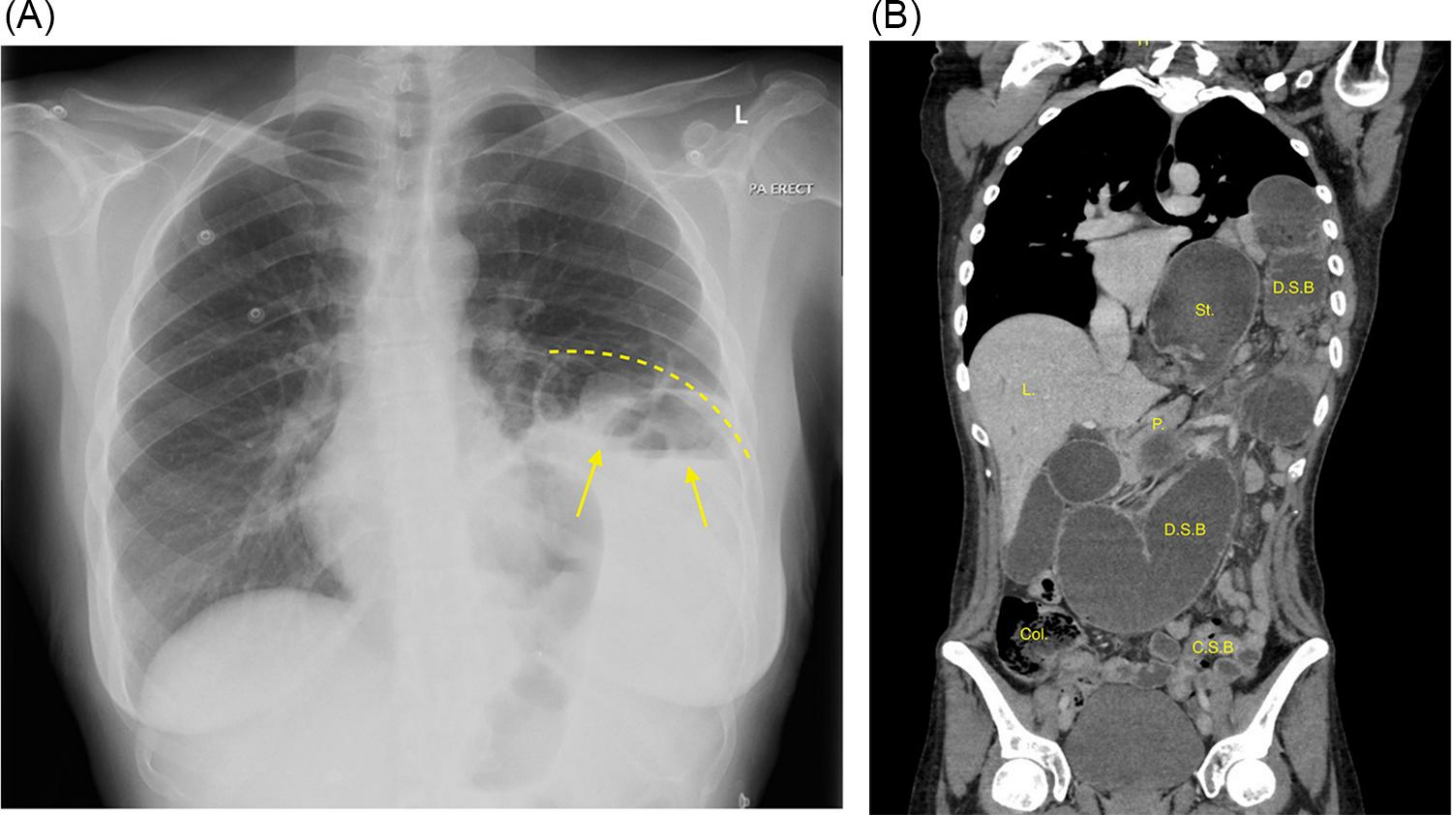
**(A)** Chest x-ray, PA view. Showing large left diaphragmatic hernia with obstruction, demonstrated by herniated small bowel loops with air-fluid levels (arrows) **(B)** CT scan of the chest, abdomen, and pelvis with contrast, coronal view. Showing large left diaphragmatic hernia containing a dilated stomach, dilated small bowel loop and collapsed small bowel loops. (L.;liver, P.;pancreas, St.;stomach, Col.;colon, D.S.B; dilated small bowels, C.S.B.; collapsed small bowels).

She was hemodynamically stable after receiving analgesics. Physical examination revealed upper abdominal tenderness with no guarding or rigidity, and bowel sounds were hypoactive. Laboratory investigations were unremarkable, with hemoglobin 12.5 g/dL, white blood cell count 9.6 × 109/L, lactate 1.4 mmol/L, amylase 64 U/L, and normal liver enzymes and renal function tests. Cardiac enzymes and electrocardiogram were normal, excluding acute coronary syndrome. Chest x-ray revealed a large left diaphragmatic hernia with evidence of obstruction, demonstrated by herniated small bowel loops with air-fluid levels (Figure 3A). Contrast-enhanced CT revealed a large left diaphragmatic hernia containing a dilated stomach, dilated small bowel loop, and collapsed small bowel loops. Suggesting a strangulated hernia (Figure 3B). Differentiating between partial obstruction and full strangulation was critical for determining the urgency of surgical intervention. Other potential causes of acute upper abdominal pain, such as peptic ulcer disease, acute pancreatitis, cholecystitis, and bowel obstruction unrelated to hernia, were considered but subsequently ruled out based on imaging findings and laboratory results, including unremarkable liver enzymes and amylase levels.

**Figure 4:**
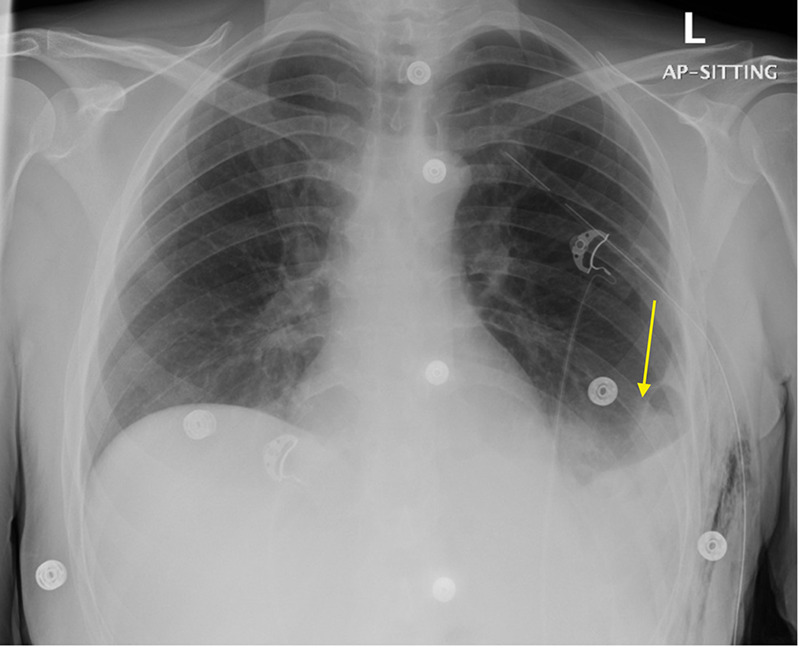
Postoperative chest x-ray, AP view. Showing reduced previously herniated abdominal organ **(arrow)**.

The patient underwent emergency exploratory laparotomy under general anesthesia. Intraoperatively, a diaphragmatic defect measuring 12 × 6 cm was identified. The herniated contents were successfully reduced, and the defect repaired using a polypropylene mesh secured with interrupted nonabsorbable sutures. No bowel resection was required. The operative time was around 150 min, with an estimated blood loss of 200 mL. The image findings and intraoperative location of the defect were consistent with a left-sided Bochdalek hernia. Given her previous abdominal surgeries, an acquired diaphragmatic injury was considered; however, the absence of scarring or evidence of prior injury to the diaphragm, along with the posterolateral position of the defect, favored a congenital etiology. Postoperatively, she developed minimal left-sided pneumothorax with mild surgical emphysema, which resolved spontaneously. The intercostal drain was removed on postoperative day six. She was discharged three days later in stable condition. At one-month follow-up, she reported resolution of pain and a return to normal activities, and subsequent chest x-rays confirmed successful hernia reduction (Figure 4).

## Patient perspective

The patients provided their perspectives on the treatments they received, reflecting their experiences and satisfaction with care. The first patient, who underwent elective diaphragmatic hernia repair despite being asymptomatic, expressed relief knowing the hernia had been repaired and was satisfied with the surgical outcome, noting no new symptoms and a return to normal activities. The second patient, who presented acutely and underwent emergency surgery, reported initial anxiety and discomfort due to the sudden onset of symptoms but felt grateful for the timely intervention, noting gradual improvement in pain, breathing, and gastrointestinal function after recovery. Both patients appreciated the explanations provided by the surgical team, felt involved in the decision-making process, and expressed confidence in the care received. Each patient provided written consent to have their cases documented for publication.

## Discussion

Congenital diaphragmatic hernia (CDH) in adulthood is exceedingly rare, with an incidence of 0.17% (3), yet its discovery carries significant clinical implications due to the risk of complications such as obstruction, strangulation, or respiratory compromise. The cases presented illustrate the variability in presentation: one patient was completely asymptomatic and diagnosed incidentally, while the other presented with acute abdominal pain, nausea, vomiting, and dyspnea.

The etiology of CDH remains largely attributed to failure of diaphragmatic closure during fetal development (6), though environmental factors such as vitamin A deficiency and exposure to teratogenic drugs, including thalidomide and anticonvulsants that disrupt purine biosynthesis, may contribute (6). Sporadic cases predominate; however, familial inheritance patterns including autosomal recessive, autosomal dominant, and X-linked have been reported (7). Adult-onset diaphragmatic hernias may also be acquired, commonly following trauma. Evidence suggests that 12% to 66% of traumatic diaphragmatic injuries are initially undiagnosed, often due to their rarity and the presence of concomitant injuries (8).

While a minority of patients present with completely asymptomatic hernias, often diagnosed incidentally through CT scans conducted for unrelated reasons, the majority (86%) report symptoms related to CDH (9). These symptoms include shortness of breath, chest discomfort, food intolerance, gastroesophageal reflux, nausea, vomiting, abdominal distension, and abdominal pain—the latter being the most commonly reported (6). In cases of congenital hernias that remain undetected into adulthood, they may stay clinically silent unless abrupt alterations, such as strangulation or respiratory compromise, occur (8).

Diagnosis in adults relies heavily on imaging, with chest x-ray serving as an initial study and CT scans providing definitive evaluation. MRI and ultrasonography may be used in selected cases (6). Radiographic misinterpretation is possible, as hernias may mimic mediastinal masses, pericardial fat pads, or other thoracic pathologies (10, 11). Bochdalek hernias most commonly involve herniation of the colon, small bowel, liver, and kidney, though rare involvement of other organs such as the gallbladder or pancreas has been reported (12).

Historically, surgical repair has been advised for all diaphragmatic hernias to prevent the risk of tissue strangulation. In asymptomatic presentations, however, the decision to operate must be individualized, considering patient characteristics such as age, activity level, overall health status, patient preference, and the specific anatomy of the hernia. For example, a young, otherwise healthy individual faces a higher lifetime risk of complications; therefore, preventive surgery is often recommended. Conversely, an elderly or frail patient may not be an ideal surgical candidate, as operative risks may outweigh potential benefits, particularly if the patient leads a sedentary lifestyle. In such cases, conservative management may be more appropriate.

Occupation is another critical factor influencing management. Jobs involving frequent alterations in intra-abdominal or intrathoracic pressure—such as diving, firefighting, military service, or professional sports—pose a higher risk for complications compared to desk-based occupations. Additionally, the size of the diaphragmatic defect and the type and number of herniated organs influence decision-making; small defects containing only omental fat can often be observed safely without surgery. Previous reports have described conservatively managed right-sided Bochdalek hernias with an intrathoracic ectopic right kidney, in which the patient remained asymptomatic over an 18-month follow-up period (12). In our report, one of the cases highlights this principle, as the asymptomatic hernia could theoretically have been managed conservatively; however, surgical repair was pursued to mitigate future risk given the patient's young age and occupational exposure.

As a final note, the decision to operate or conservatively manage an asymptomatic diaphragmatic hernia depends on the predicted risk of future complications, which must be weighed against the patient's individual circumstances. A key question remains: is conservative management simply a temporary observation period until complications arise, or can patients live their normal life expectancy without hernia-related issues? This remains challenging to answer due to the rarity of adult CDH, the generally short follow-up periods reported in the literature, and the fact that conservative management is not the principal treatment modality.

While laparotomy remains, the predominant surgical method employed (38%), minimally invasive techniques have gained traction since their introduction in 1995. Laparoscopic repair has demonstrated a low complication rate (7%), along with shorter hospital stays and faster recovery, averaging four days. According to Bridges and Hasson (13), the evolution of minimally invasive surgery has redefined the management of adult congenital diaphragmatic hernias, with laparoscopic repair now favored for its superior visualization of the diaphragmatic defect, reduced postoperative pain, and lower risk of respiratory complications compared to open approaches. These advantages are particularly significant in hemodynamically stable patients without extensive visceral herniation (13). Following reduction, diaphragmatic defects are typically repaired via primary closure or mesh reinforcement. Mittal et al. (10) emphasize that tension-free closure is essential to prevent recurrence, recommending mesh reinforcement for defects measuring ≥5 cm. In their case series, laparoscopic mesh repair resulted in favorable outcomes, minimal postoperative pain, and early mobilization. Larger defects (>10 cm) may necessitate muscle flap reconstruction prior to mesh reinforcement to provide additional structural support and prevent excessive tension at the suture line (10). These findings underscore the adaptability of minimally invasive techniques in managing both acute and chronic presentations.

Conversion to open surgery remains an important operative metric, reflecting the technical complexity and intraoperative challenges often encountered. Although data specific to adult BH are limited, broader analyses of diaphragmatic hernia repairs report conversion rates ranging from 0% in highly selected elective cases to as high as 24.3% in mixed adult cohorts. In a retrospective analysis of 70 adult diaphragmatic hernia repairs, 60% were performed laparoscopically, with a 24.3% conversion rate to open surgery [Köneş et al. (14)]. In contrast, small series have reported no conversions in selected cases managed with laparoscopic primary suturing and mesh reinforcement [Palanivelu et al. (15)], demonstrating the feasibility of minimally invasive repair in properly chosen patients. Although higher conversion rates can be observed in more complex or chronic diaphragmatic hernias, it likely reflects variations in case selection, defect chronicity, and institutional experience.

A thoracic approach, while more invasive, may be indicated in cases with significant fibrosis or dense adhesions—common in chronic or delayed presentations—where visualization and safe dissection from the thoracic cavity are more achievable. Starr et al. (8) reported that the choice between thoracic and abdominal (laparoscopic or open) approaches depends on several patient-specific factors, including chronicity, extent of organ involvement, and the presence of complications such as obstruction or strangulation. Additionally, institutional capability and surgeon expertise remain crucial determinants of approach selection (8).

These trends are reflected in a recent systematic review of adult right-sided Bochdalek hernias, which analyzed surgical approaches and outcomes. A systematic review was conducted on adult right-sided Bochdalek hernias, excluding those that were left-sided, pregnancy-associated, pediatric, or traumatic. Right-sided diaphragmatic hernias are less common than left-sided ones due to the earlier closure of the right canal and the protective effect of the liver. A total of 401 cases were identified in the records, and only 44 adult cases were included after screening. The mean age of the patients was 58 years, with two peaks in distribution: 40–50 years and 70–80 years. It was more prevalent among females (61%) than males (39%). The most common symptom was dyspnea, observed in about half of the patients, followed by abdominal pain (43%). Other reported symptoms included chest pain, nausea, and shoulder or flank pain. CT scan was the diagnostic method in 89% of cases. The reported defect size in 23 cases ranged from 0.5 × 0.3 cm to 12 × 10 cm. The organs most frequently identified in the herniation were colon (52%), small bowel (43%), liver (27%), and kidney (18%), with some cases involving the gallbladder or pancreas. Majority of cases (86%) underwent surgical repair. The surgical approach varied: abdominal laparotomy (34%), laparoscopy (34%), thoracotomy (16%), thoracoscopy (14%), and some with robotic assistance. The method of defect repair also differed: direct suture (45%) and mesh-augmented repair (43%). Twenty-five patients (57%) did not experience any complications related to the hernia or intervention. Reported complications primarily included hydronephrosis (*n* = 4, 9%) due to ureteral or renal involvement, thoracic complications such as abscesses and empyema (18%), and bowel ischemia or perforation in a few cases. About 9% of patients died postoperatively due to pneumonia, sepsis, bowel perforation, or cerebrovascular events. A key clinical takeaway is that although Bochdalek hernias are rare, they often present as acute surgical emergencies. The surgical approach should be determined on a case-by-case basis depending on the presence of visceral complications. For repair, non-absorbable mesh and sutures are preferred, when possible, but mesh should be used with caution if contamination is present. Follow-up is recommended for up to 10 years (16). A summary table of findings is shown below (Table 1).

**Table 1:** Summary of Key findings—ramspott et al. (16).

Category	Details
Included Patients	401 cases identified; 44 adult patients included
Mean age/Range	58 years (22–92); two peaks: 40–50 yrs and 70–80 yrs.
Sex	61% female (27); 39% male (17).
Symptoms	Dyspnea ≈ 50%, abdominal pain ≈ 43%, chest/flank pain ≈ 18%.
Imaging used	CT 89%; x-ray 4%; MRI 5%.
Herniated organs	Colon 52%, small bowel 43%, liver 27%, kidney 18%.
Defect size	Reported in 23 cases: range 0.5 × 0.3 cm to 12 × 10 cm.
Surgical treatment	86% (38/44) underwent surgery
Approach	Laparotomy 34%; laparoscopy 34%; thoracotomy 16%; thoracoscopy 14%; robotic ≈ 5%.
Repair method	Suture 45%; mesh 43%; suture + mesh 16%; non-absorbable mesh most common.
Complications	None 57%; thoracic 18% (abscess/empyema/pneumonia); hydronephrosis 9%; bowel ischemia/perforation ≈ 4%.
Mortality	Mortality 9% (4 cases); median follow-up 9 months; no recurrences reported.

Furthermore, Ramspott and colleagues note persistent gaps in evidence concerning the necessity of repair in asymptomatic patients, timing of intervention, long-term outcomes, and comparative efficacy of laparoscopic vs. thoracoscopic approaches (12). As minimally invasive modalities continue to evolve, future research should focus on standardizing indications and defining patient profiles most likely to benefit from laparoscopic repair, balancing operative safety with long-term functional outcomes.

Long-term data on adult CDH repair remain limited because of its rarity, but available studies suggest that recurrence is uncommon, especially when mesh reinforcement is used (13, 16). Bridges and Hasson (13) found no late recurrences during follow-up periods of up to five years, and Ramspott et al. (16) reported that most patients maintained good postoperative quality of life with few or no residual symptoms. However, both studies note that long-term evidence is scarce and that more consistent follow-up and reporting are needed to clarify outcomes and recurrence risks over time.

In the first case presented, the patient was asymptomatic, and the diaphragmatic defect was discovered incidentally. Due to limited intraoperative visualization, the procedure was converted to open laparotomy, and mesh reinforcement was used for a defect >5 cm. The patient recovered well postoperatively despite developing a pneumothorax.

In the second case, the patient presented acutely with abdominal pain, nausea, vomiting, and shortness of breath. Emergency laparotomy revealed a 12 × 6 cm diaphragmatic defect containing the stomach, small bowel, and transverse colon. The defect was repaired using mesh, and although the patient developed a postoperative pneumothorax, overall recovery was uneventful.

These cases underscore that adult CDH may present variably from incidental findings to acute surgical emergencies and management must be tailored based on patient characteristics, hernia anatomy, and potential risk of complications. Early recognition, careful surgical planning, and individualized decision-making are critical to optimizing outcomes.

## Conclusion

Adult congenital diaphragmatic hernias are rare and may present either incidentally or with acute symptoms. Diagnosis relies primarily on CT imaging, as chest x-rays may fail to detect or fully characterize the hernia. Surgical repair remains the definitive treatment, as delaying intervention increases the risk of complications such as organ strangulation or respiratory compromise. The choice of surgical approach should be individualized, considering patient factors, hernia size, and the type and number of herniated organs. This report presented two adult cases with distinct presentations: one discovered incidentally with significant herniation into the thorax and mediastinal shift, and the other symptomatic but hemodynamically stable. Both cases underwent successful surgical repair, highlighting the importance of tailored management and multidisciplinary planning in optimizing patient outcomes.

## Data Availability

The raw data supporting the conclusions of this article will be made available by the authors, without undue reservation.
